# Limited decadal growth of mountain birch saplings has minor impact on surrounding tundra vegetation

**DOI:** 10.1002/ece3.9028

**Published:** 2022-06-23

**Authors:** Ruud Scharn, Isabel S. Negri, Maja K. Sundqvist, Jørn O. Løkken, Christine D. Bacon, Alexandre Antonelli, Annika Hofgaard, R. Henrik Nilsson, Robert G. Björk

**Affiliations:** ^1^ Department of Earth Sciences University of Gothenburg Gothenburg Sweden; ^2^ Gothenburg Global Biodiversity Centre Gothenburg Sweden; ^3^ School of Biosciences Cardiff University Cardiff UK; ^4^ Department of Forest Ecology and Management Swedish University of Agricultural Sciences Umeå Sweden; ^5^ Norwegian Institute for Nature Research Trondheim Norway; ^6^ Department of Biology Norwegian University of Science and Technology Trondheim Norway; ^7^ Department of Biological and Environmental Sciences University of Gothenburg Gothenburg Sweden; ^8^ Royal Botanical Gardens Kew Richmond UK; ^9^ Department of Plant Sciences University of Oxford Oxford UK

**Keywords:** *Betula pubescens*, climate change, Oroarctic, phylogenetic diversity, plant community structure, treeline advance

## Abstract

Temperatures over the Arctic region are increasing at three times the rate of the global average. Consequently, Arctic vegetation is changing and trees are encroaching into the tundra. In this study, we examine the establishment and growth of mountain birch (*Betula pubescens* ssp. *tortuosa*), which forms the treeline in subarctic Europe, and its impact on community composition across the treeline ecotone nearby Abisko, Sweden. Birch advancement along elevational gradients was studied by comparing data collected in 2016 with data collected 10 and 15 years previously. Species identity, cover, and phylogenetic relatedness were used to assess the impact of birch encroachment on community composition. Our results show that birch occurrence above the treeline did not affect plant community composition, probably owing to the observed lack of significant growth due to herbivore browsing, nitrogen limitation, or a reduction in snow cover. Independent of birch performance, the tundra community structure shifted toward a novel community dissimilar from the forest plant community found below the treeline. Taken together, our findings are explained by species‐specific responses to climate change, rather than by a linear forest advance. Future treeline advancements are likely more restricted than previously expected.

## Graphical abstract

Mountain birch has minor impact on surrounding tundra vegetation. Nonetheless tundra vegetation is shifting, not towards forest vegetation, but towards an alternative vegetation state.

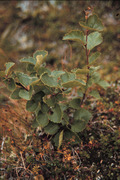

## 1 INTRODUCTION

Climate change has major impacts on Arctic environments. Compared with lower latitudes, average air temperatures have been rising at three times the rate in Arctic and Polar Regions since 1971 (AMAP, 2021). In the past few decades, Arctic summers have been the warmest in 2000 years, and average autumn–winter temperatures in northern areas are expected to increase by between 3°C (SSP1‐2.6) and 12°C (SSP5‐8.5) by 2081–2100 (Lee et al., 2021). Consequently, vegetation changes observed in response to recent warming include an increase in primary productivity (Arctic greening) and in the cover of shrubs and trees (e.g., Bjorkman et al., 2018; Elmendorf et al., 2012; Myers‐Smith et al., 2020). The taller vegetation as a result of the increase in productivity remains more exposed above the snow cover in winter and hence reduces surface albedo (Bjorkman et al., 2018; Sturm et al., 2001). Models have shown a reduction in winter albedo of almost 2% between 1980 and 2006 due to longer growing seasons (Bonfils et al., 2012). A decline in snow cover, coupled with a decrease in albedo due to forest expansion, can accentuate further warming to such an extent that it overrides the negative climate feedback resulting from increased carbon uptake by trees (Betts, 2000; de Wit et al., 2014). Thus, the expansion of trees and shrubs into the Arctic is of urgent global concern (Larsen et al., 2015).

Shifts in treeline position uphill or poleward into tundra ecosystems under climate change have been observed in many locations around the world (e.g., Bryn & Potthoff, 2018; Harsch et al., 2009; Rees et al., 2020). Globally, 52% of all treelines studied have advanced, with only 1% reporting treeline recession (Harsch et al., 2009). Particular treelines in areas experiencing strong winter warming were more likely to advance (Harsch et al., 2009). In addition, the altitudinal position of the treeline has been found to positively correlate with a ground temperature of 6–7°C in the mean growing season (Körner & Paulsen, 2004), which suggests that the treeline positions are sensitive to a changing climate. Climate scenarios of future treeline advance in the Abisko region of northern Sweden predict the treeline to advance between 45 (RCP 2.6) and 195 (RCP 8.5) elevational meters by the year 2100 (Gustafson et al., 2021).

Despite strong global evidence for climate‐driven treeline advance, scale‐dependent factors such as varying thermal conditions, soil type, and nutrient availability can have a strong influence on local patterns in treeline advance (Holtmeier & Broll, 2017). In addition, mammal browsing and more local patterns of herbivory (e.g., by invertebrates) can restrict tree advancement into the tundra (Kaarlejärvi et al., 2013; Van Bogaert et al., 2011). Due to large regional variation in these variables (Hofgaard et al., 2012), studies have identified slow advance (Mathisen et al., 2014; Rees et al., 2020), retreat (Dalen & Hofgaard, 2005), stability in distribution (Hofgaard et al., 2009), and increased forest density (Lescop‐Sinclair & Payette, 1995) in forests within the same region. Tree species in the Swedish Scandes have advanced uphill in recent decades (Kullman, 2010), where, for instance, in the year 2000, mountain birch saplings were identified almost 400 m higher than the uppermost limit for this species recorded in 1955 (Kullman, 2002). Though the establishment of these birch saplings is restricted to local enclaves with a more favorable microclimate, they do not necessarily indicate treeline advance (Løkken et al., 2020; Sundqvist et al., 2008). The plant community composition in the direct vicinity of birch individuals that have been established on the tundra was also found to shift by becoming more similar to the composition in the nearby birch forest (Sundqvist et al., 2008). This may indicate that mountain birch has a positive effect on the growth of some subalpine species, whereby birch saplings ameliorate the habitat and buffer some neighboring plants from open tundra‐related stress (Bertness & Callaway, 1994; Brooker et al., 2008). For example, mature mountain birches have been shown to facilitate birch seedling performance and survival at high‐stress sites with strong winds and extreme temperatures (Eränen & Kozlov, 2008). They have furthermore been found to facilitate the establishment of other trees, such as Scots pine (Mikola et al., 2018). In addition, both warming and mountain birch leaf litter can increase moss‐associated nitrogen fixation in subarctic tundra environments, promoting shrub expansion (Rousk & Michelsen, 2017).

Here, we study the growth of mountain birches that have been established in the Swedish tundra for over 15 years (Sundqvist et al., 2008; Truong et al., 2007), and explore how alpha (*α*) diversity and beta (*β*) diversity have varied over time in tundra communities with and without birch saplings. Further, we compare diversity patterns in local tundra communities with and without mountain birch with those in a nearby mountain birch forest. Based on previous observations (Sundqvist et al., 2008), we predict that birch‐infiltrated tundra communities have become more similar to birch forest communities in terms of their *α*‐ and *β*‐diversity over time. Finally, we assess whether these patterns are consistent over multiple aspects of diversity by comparing species richness, evenness, and phylogenetic diversity.

## 2 MATERIALS AND METHODS

### 2.1 Study sites

**FIGURE 1:**
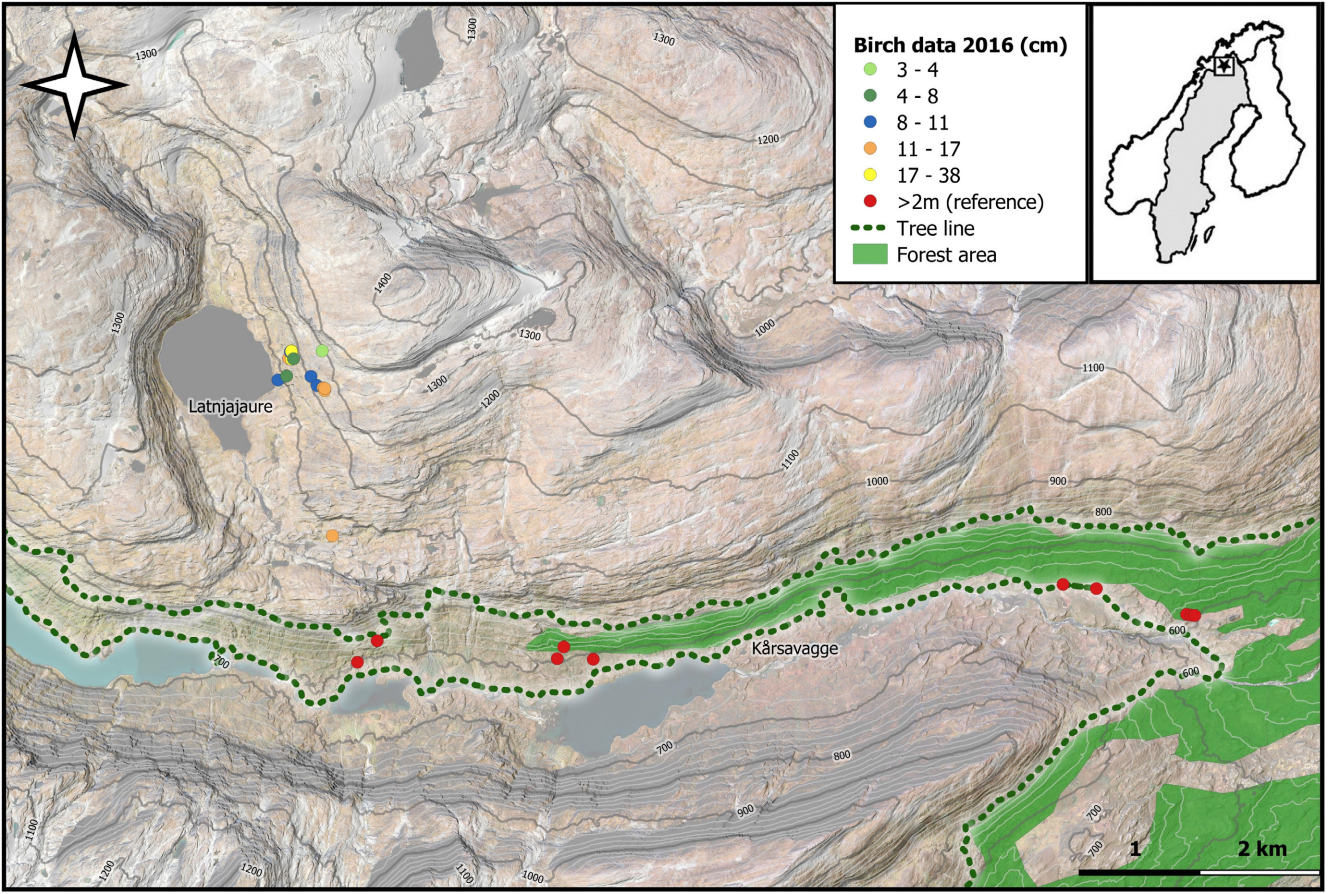
Elevation map over Latnjajaure and Kårsavagge derived from ground elevation model at 1‐m grid cell size (Lantmäteriet, 2019a). Locations and heights of birch saplings included in the study are marked by dots. Solid lines and green shading mark the treeline and closed forest area, respectively. Both were derived from a synthesis of RGB orthophoto (Lantmäteriet, 2019b), the Swedish National Land Cover Database (Naturvårdsverket, 2020), and field data

The study area is located in the area around Latnjajaure Field Station (68°21′ N, 18°30′ E) at the shoreline of lake Latnjajaure (990 m a.s.l.).The closest forest in this area is located in the lower valley of Kårsavagge south of Latnjajaure (610–810 m a.s.l.), northern Swedish Lapland (Figure 1). The mean annual temperature at Latnjajaure Field Station ranges from −2.9°C to −0.5°C (1993–2016) and has increased at a rate of 0.4°C [CI = 0.2–0.7°C] per decade from 1993 to 2016 (Scharn, Brachmann, et al., 2021). The mean annual precipitation is 847 mm, with a range of 605 and 1091 mm (1990–2015; Scharn, Brachmann, et al., 2021). There is no in‐site climate data for the forest, but at the nearby Abisko meteorological station (385 m a.s.l.), the mean annual temperature is +0.3°C (1991–2011), with an increase of 2.6°C from 1913 to 2006 (Callaghan et al., 2013). The mean annual precipitation is 332 mm (2002–2011; Callaghan et al., 2013). The Latnjavagge valley is an Oroarctic tundra (Virtanen et al., 2016) covered by snow from October to mid‐June most years. Vegetation composition in the valley varies depending on altitude, soil moisture, and the time of final thaw, as well as soil nutrients and pH (Lindblad, 2006). The area is grazed by mountain hare (*Lepus timidus*), rock ptarmigan (*Lagopus mutus*), lemming (*Lemmus lemmus*), and semi‐domesticated reindeer (*Rangifer tarandus*). Reindeers graze in the area from July to September with a density of approximately 2.3 reindeers/km^2^ (Vowles et al., 2016).

The treeline reaches about 810 m a.s.l. (where the uppermost 2‐m tall mountain birch tree has been found; Sundqvist et al., 2008). The upper range of the closed birch forest in the area is at around 700 m a.s.l., but at Latnjajaure, 100 birch saplings have been recorded above the treeline within an altitudinal range of 810–1170 m a.s.l. (Sundqvist et al., 2008; Truong et al., 2007). The growth (height, number of leaves, and number of branches) of these birch saplings above the treeline has been found to be positively correlated with abiotic factors such as southern exposure resulting in higher air temperatures and longer growing season due to earlier snowmelt (Truong et al., 2007). Further, at approximately 1060 m a.s.l., around 30 saplings have been established within a 10‐m radius of each other in an area, which, due to local topography, has a warmer microclimate than the valley floor near the Latnjajaure Field Station. Most of the mountain birch saplings in Latnjajaure are situated on a southwest‐facing slope, where soil pH and nutrient content are high, and where Garnet mica schists with some inclusions of marble and dolomite dominate the bedrock (Kulling, 1964).

### 2.2 Data collection

In July 2016, we revisited birch individuals previously observed by Sundqvist et al. (2008) in Latnjavagge in 2006. Seven of 18 individuals from the previous dataset were found and included, supplemented by nine individuals that were identified in this study. Hence, in total 16 individuals were measured above the treeline at elevations of 960–1180 m a.s.l. A 50 × 50 cm plot was placed around each birch individual (named “Betula” plots), and Control plots were assigned approximately 10 m away from each “Betula” plot following the methodology in Sundqvist et al. (2008). For each plot (Betula and Controls), all vascular plant species were recorded and plant cover was estimated through the Braun–Blanquet method (Braun‐Blanquet, 1932). As the coordinates of the 2006 Reference plots were unavailable, in 2016 thirteen new “Reference” vegetation plots were established at Kårsavagge from the treeline at 810 m a.s.l. to the closed forest at 610 m a.s.l. close to where birch trees were present, following the methodology in Sundqvist et al. (2008). Height, base diameter, and number of stems were measured for all the birch saplings. Additionally, in the same area we measured the 12 birch individuals marked and measured in 2001 by Truong et al. (2007) to estimate their growth over our study period as neither height nor diameter was measured on the birches observed by Sundqvist et al. (2008) in 2006.

### 2.3 Phylogeny

We used the time‐calibrated phylogenetic tree of vascular plants in Latnjajaure generated by Scharn, Little, et al. (2021). We pruned the tree to only contain species observed in this study using the package ape (v5.3; Paradis et al., 2004) in the R programming language (version 4.0.0; Arbor Day, R Core Team, 2018).

### 2.4 Diversity metrics

For each plot, we calculated species richness (SR) as the total number of each species in each plot, and Simpson's index of diversity (D), where an increase in this index represents an increase in diversity (Magurran, 2003). Based on the maximum‐likelihood tree obtained from the phylogenetic inference, *α‐* and *β*‐diversity indices were calculated using the interMPD (interspecific abundance‐weighted mean pairwise distance; Miller et al., 2017) and the netMPD (abundance‐weighted net difference in between plot MPD; Scharn, Little, et al., 2021), respectively. Both measures resulted in a clustering value representing mean evolutionary time (measured in millions of years) between individuals within plots in the former case, and between plots in the latter (Miller et al., 2017; Scharn, Little, et al., 2021). In order to measure *β*‐diversity based on pure presence/absence, as well as abundance‐weighted input, we used Jaccard and Bray–Curtis dissimilarity indices, respectively.

### 2.5 Statistical analyses

All analyses described below were done in R (version 4.0.0; Arbor Day, R Core Team, 2018), using the following packages: *ggplot2* (v3.3.2; Wickham, 2016), *vegan* (v2.5‐6; Oksanen et al., 2016), *rjags* (v4‐10; Plummer, 2016), and *R2jags* (v0.6–1; Su & Yajima, 2015).

Height (cm) and diameter (mm) of permanently marked birches at Latnjajaure were compared with data collected in 2001 by Truong et al. (2007) using a generalizable Bayesian modeling approach. The model included either height or diameter as its dependent variable, year as a fixed effect, and altitude (factor) as a random effect (see below). All Bayesian models utilized Markov chain Monte Carlo with two chains each consisting of 30,000 iterations of which the first 20,000 were discarded as burn‐in. We define a “significant” effect where the difference between categories did not overlap with 0 in its 95% credible interval (CI). We used noninformative priors for all coefficients and checked for convergence of the chains for all parameters using the Gelman–Rubin convergence statistic (Gelman & Rubin, 1992).

$$\text{BirchHeight}\sim \text{normal}\left(\mu_{y}+\sigma_{0}\right)$$

$$\mu_{y}\sim \text{normal}\left(\alpha_{y}+\alpha_{a},\sigma_{1}\right)$$

where *μ*_*y*_ = mean value for each community year, *α*_*y*_ = intercept value for a certain community and year, *α*_*a*_ = altitude‐level (factor) random effect, *σ* = the variance parameter of the normal distribution, and μα = the intercept mean.

To estimate the variation of each diversity metric between community types and years, we used a similar model to the birch height model described above. The model included diversity metrics as a dependent variable and community, year, and their interactions as fixed effects. The example below describes the model used to model interMPD. The models used to describe SR and D responses follow the Poisson and beta error distributions, as they represent count and 0–1 bounded data, respectively, but are otherwise identical.

$$D_{\mathrm{mpd}}\sim \text{normal}\left(\mu_{c,y}+\sigma_{0}\right)$$

$$\mu_{c,y}\sim \text{normal}\left(\alpha_{c,y}+\alpha_{a},\sigma_{1}\right)$$

where *D*_mpd_ = the observed interMPD per plot, *μ*_*c,y*_ = mean value for each community and year, *α*_*c,y*_ = intercept value for a certain community and year, *α*_*a*_ = altitude‐level (factor) random effect, *σ* = variance parameter of normal distribution, and μα = the intercept mean. The models also included altitude as a random effect to account for the nonindependence of plants measured within the same altitude and the same plot measured in both years.

In addition to the intercept‐only model, we tested whether community differences varied with altitude. Similar to the intercept‐only model, the linear model included diversity metrics as a dependent variable and community, year, and their interactions as fixed effects using altitude as a linear predictor rather than as a random effect.

The example below describes the model used to model interMPD. The models used to describe species richness and Simpson's responses follow the Poisson and beta error distributions, as they represent count and 0–1 bounded data, respectively, but are otherwise identical.

$$D_{\mathrm{mpd}}\sim \text{normal}\left(\mu_{c,t,g,y}+\sigma_{0}\right)$$

$$\mu_{c,t,g,y}\sim \text{normal}\left(\alpha_{c,y}+\beta_{c,y}+A_{a},\sigma_{1}\right)$$

where *D*_mpd_ = the observed mean pairwise distance (interMPD) per plot, *μ*_*c,y*_ = mean value for each community and year, *α*_*c,y*_ = intercept value for a certain community and year, β_*c,y*_ = slope value for a certain community and year over altitude $\left(A_{a}\right)$, *σ* = variance parameter of normal distribution, and μα and μβ represent the intercept and slope means, respectively.

We used the betadisper function in vegan (Anderson, 2006) to calculate and compare within‐community *β‐*dispersion using Jaccard, Bray–Curtis, and netMPD indices among both years of measurement. We then compared taxonomic community composition through time using permutational multivariate analysis of variance (PerMANOVA) implemented with the Adonis function in vegan, constraining the permutations to each unique altitude to account for repeated sampling through time (Anderson, 2001). We used nonmetric multidimensional scaling (NMDS) to identify patterns between communities and over time. Confidence interval ellipses and community centroids were extracted using the ordiellipse function in the R package vegan (Kruskal, 1964; Minchin, 1987).

## 3 RESULTS

### 3.1 Birch growth

Among all 28 birches measured above the treeline in 2016, average height was 14 cm (ranging from 3.5 to 37.5 cm) and average base diameter was 7 mm (1.2 mm ‐ 32.5 mm). Birches showed no significant increase in height [CI = −4.6 cm, 7.9 cm] compared with those in 2001, but base diameter had increased [CI = 0.7 mm, 3.8 mm] over the same time span.

### 3.2 Species turnover between years

In 2006, we recorded an average of 11.69 species in Control, 11.89 species in Betula, and 11.26 species in Reference plots. In 2016, we recorded 11.51, 11.40, and 11.14 species for Control, Betula, and Reference plots, respectively. Average percent coverage of selected species within plots in 2016 revealed that *Vaccinium myrtillus* had established in the Control plots. In addition, *Avenella flexuosa*, *Diphasiastrum alpinum*, and *Carex concolor—*species that had not been observed in previous measurements—appeared in 2016 and were established mainly in Betula plots. Furthermore, *Carex bigelowii* (−69%), *Thalictrum alpinum* (−94%), and *Bistorta vivipara* (−68%) had decreased in cover relative to 2005/2006, with *C*. *bigelowii* (−32%), *T*. *alpinum* (−96%), and *B*. *vivipara* (−88%) all decreasing the most in Control plots. Similar to 2006, *Phyllodoce caerulea* (+44%) and *Empetrum nigrum* ssp. *hermaphroditum* (+50%) had a higher cover in Betula than in Control plots in 2016, although *E*. *nigrum* ssp. *hermaphroditum* had decreased in 2016 in both Betula (−71%) and Control (−21%) plots. One species found at higher elevations in 2016 than in 2006 was the predominantly boreal *Trientalis europaea* that was observed growing in a Control plot at 1100 m a.s.l. in 2016, while it was observed at 990 m a.s.l. in 2006.

### 3.3 Birch effect on plant diversity

**FIGURE 2:**
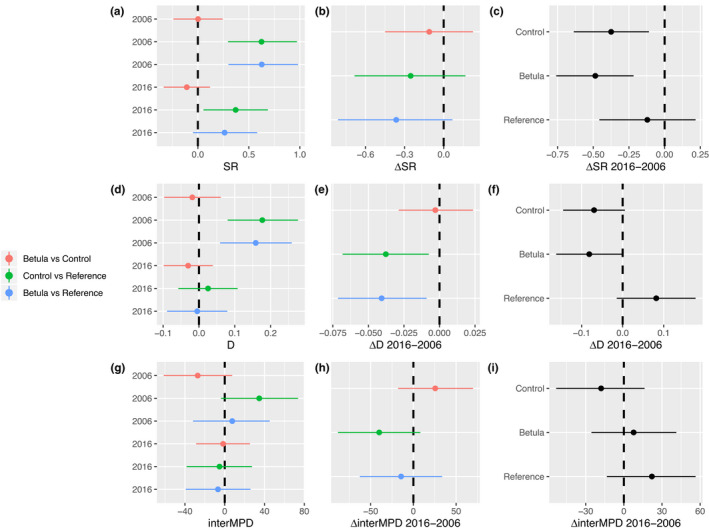
Difference within diversity indices species richness (SR), Simpson's diversity index (D), and mean pairwise difference (interMPD) between the Control, Betula, and Reference communities between 2006 and 2016. Subplots show the difference in diversity between communities within years (a, d, and g), the change in these diversity differences between years (b, e, and h), and the difference within communities over time (c, f, and i). Dots represent estimate means with vertical lines that span the 95% credible intervals for each estimate

**FIGURE 3:**
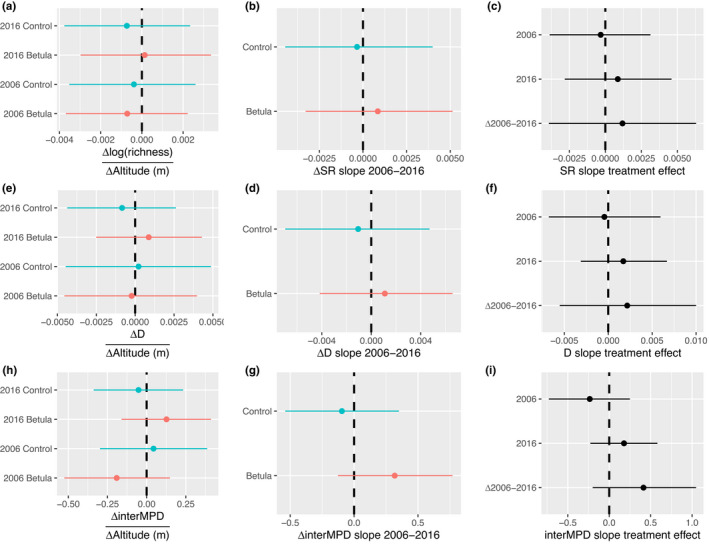
Community difference over altitude within diversity indices species richness (SR), Simpson's diversity index (D), and mean pairwise difference (interMPD) between the Control, Betula, and Reference communities between 2006 and 2016. Subplots show the slope per community within each year (a, d, and g), the difference in diversity change over altitude within treatments between years (b, e, and h), and the difference in diversity change over altitude between communities within years (c, f, and i). Points represent the mean, and vertical lines span the 95% credible intervals for each estimate

A total of 75 vascular plant species were identified across all plots at Latnjajaure in 2016 (73 in 2006). α‐Diversity measurements of SR and D showed a similar pattern where both the Betula and Control plots were significantly more diverse than Reference plots in 2006, but not from each other (Figure 2a,d). In 2016, the Control plots were significantly more species‐rich than the Reference plots (Figure 2a). Between 2006 and 2016, the differences in D, but not in SR, between the tundra plots and Reference plots decreased (Figure 2b,e). This was driven by a contrasting effect of the evenness component of D (Figure 2f) where tundra plots (i.e., both Betula and Control plots) showed decreasing trends, contrasting the Reference plots, which showed an increasing trend in D. For SR, there was no difference between the responses of the Control and Reference plots (Figure 2c). No significant shift in interMPD was found between communities nor within communities over time (Figure 2g–i). In addition, we did not observe any difference in diversity over altitude between communities nor between years for any of the metrics (Figure 3).

**FIGURE 4:**
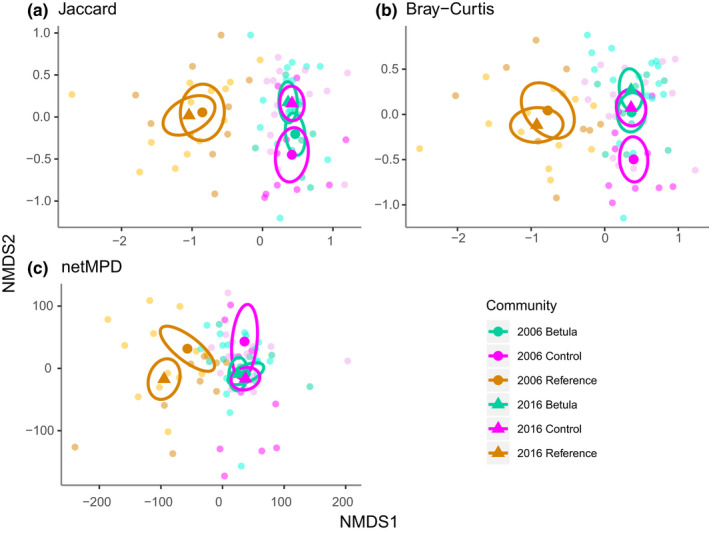
Nonmetric multidimensional scaling analyses of Betula, Control, and Reference communities using three diversity indices (a: Jaccard (*s* = 0.18, *k* = 2), b: Bray–Curtis (*s* = 0.22, *k* = 2), and c: net mean pairwise distance (*s* = 0.20, *k* = 2)). Measurements are colored by community type with large dots and triangles representing their community means during 2006 and 2016, respectively, while ellipses denote their standard deviations. The faded smaller dots represent the positions of the original plots

Results of the *β‐*dispersion analysis did not reveal any significant difference in dispersion between communities for any of the tree metrics (Jaccard, Bray–Curtis, and netMPD). When comparing community composition, however, all metrics showed significant differentiation between the Reference and both the Betula and Control communities (Figure 4). Differences between the Jaccard and netMPD metrics were only observed between the 2006 and 2016 Control plots, a trend that was also observed for the Bray–Curtis metric (Table 1).

**TABLE 1:** Results of PerMANOVA, examining the differentiation between community types and within communities but between sampling years

Comparison	*df*	*F*_stat_	*R* ^2^	*p*‐Value
*Jaccard*				
Betula 2006 × Control 2006	1,18	1.12	0.06	1.00
Betula 2016 × Control 2016	1,32	0.86	0.03	1.00
Betula 2006 × Betula 2016	1,25	1.42	0.05	.42
Control 2006 × Control 2016	1,25	3.05	0.11	<.01**
*Bray–Curtis*				
Betula 2006 × Control 2006	1,18	1.65	0.08	.36
Betula 2016 × Control 2016	1,32	1.14	0.03	1.00
Betula 2006 × Betula 2016	1,25	0.87	0.03	1.00
Control 2006 × Control 2016	1,25	2.05	0.08	.10^#^
*netMPD*				
Betula 2006 × Control 2006	1,18	1.73	0.09	.43
Betula 2016 × Control 2016	1,32	0.81	0.02	1.00
Betula 2006 × Betula 2016	1,25	0.83	0.03	1.00
Control 2006 × Control 2016	1,25	2.81	0.10	.03*

*Note*: *p* Values shown are adjusted using the Bonferroni correction. Significant results are marked with * for *p* < .05 and ** for *p* < .01, while trends (*p* < .10) are marked with ^#^.

## 4 DISCUSSION

We found that change over time rather than the presence of birches drove shifts in most *α*‐ and *β‐*diversity measures in tundra communities at Latnjajaure. Furthermore, both species richness and evenness aspects of *α*‐diversity decreased over time in the tundra sites, resulting in these communities becoming more similar to the Reference birch forest. However, in contrast to our prediction this decrease in diversity was not driven by the presence of birches. Thus, even though the birch saplings in Latnjajaure have been established for more than 15 years (Truong et al., 2007), our result points out that these mountain birches do not ameliorate their habitat enough to buffer surrounding neighbor plants from stress that would lead to a vegetation shift (Bertness & Callaway, 1994; Brooker et al., 2008). To date, studies on ameliorating effect of the presence of birches have focused on mature trees (Eränen & Kozlov, 2008; Mikola et al., 2018). Thus, the undetected impact of birch sapling presence on the surrounding vegetation may be an effect of the minimal growth we observed in these birches so far. However, they are still an important part of the sapling pool and may under more favorable conditions respond swiftly with improved growth (Kullman, 2010). Although it is unlikely that the birch saplings currently are strong drivers themselves, birch treeline advance over the past century has generally been through the rapid growth of established individuals (Kullman, 2010). Thus, if future warming increases growth, they might still be an important component in driving community change.

The limited birch sapling growth we observe was restricted to growth in stem width but not height. Air temperatures have increased in the Abisko area over the last century (Callaghan et al., 2013), but in Latnjajaure, the increase has not been as strong during the last decade as during the 1990s and early 2000s (Björk et al., 2007; Scharn, Brachmann, et al., 2021). Furthermore, the wind is causing local variation in physiognomy seen in the world's treelines (Richardson & Friedland, 2009). Thus, the windier conditions prevailing in tundra compared within the birch forest may explain the increase in stem width but not in height, and the increase in mean air temperature may yet not be enough to overcome the wind effect at this altitude. In addition, the length of the growing season has been shown to play a larger role in birch height than mean air temperature (Kullman, 2010). Hence, the prolongation of the growing season in the Abisko area (Kohler et al., 2006) may not yet have reached the threshold needed for the saplings to grow into trees. This is in line with findings that age distribution and sapling height above the treeline do not corroborate predictions of rapid forest advance in northern areas such as Abisko (Hofgaard et al., 2009). In this region, the winter is the season that is warming the most (Scharn, Brachmann, et al., 2021), which most likely does not affect birch growth itself, but may influence snow conditions. The thickness, continuity and duration, of the winter snowpack are common limiting factors for arctic vegetation as the snow provides shelter from the harsh winter climate (Wipf & Rixen, 2010), as well as protection from large herbivores such as mountain hare, ptarmigan, and reindeer (Christie et al., 2014; Nordengren et al., 2003; Rödel et al., 2004; Vowles et al., 2016). Browsing may have played a significant role in controlling birch growth (Kaarlejärvi et al., 2013; Løkken et al., 2019; Speed et al., 2011). Unlike the North American *Betula glandulosa* and *Betula neoalaskana*, *Betula pubescens* ssp*. tortuosa* is resin‐free and only produces condensed tannin as an anti‐browsing defense (Bryant et al., 2014). For this reason, it has been suggested that browsing has considerably more impact when considering *B. pubescens*, compared with that in areas such as North America, where caribou have less impact on the expansion of birches in the tundra (Bryant et al., 2014). In addition, warming has shown to not significantly affect birch saplings in the Norwegian forest–tundra ecotone, whereas browsing had a much larger impact on their growth (Hofgaard et al., 2010; Løkken et al., 2019). One alternative explanation is nitrogen limitation, which is commonly found in high latitude ecosystems (Tamm, 1991). A recent modeling study in the Abisko area suggested that nitrogen could become a limiting factor once climate conditions are favorable for growth (Gustafson et al., 2021). Nitrogen fertilization experiments have also shown that mountain birch at the treeline displays additional growth after nitrogen additions (Sveinbjornsson et al., 1992), as well as improved birch seedling survival at the treeline (Grau et al., 2012). Nitrogen is thus likely important for the establishment and growth of new individuals that drive the altitudinal advance of the treeline. Thus, mammal browsing, nitrogen limitation, a reduction in winter snow cover, or interactions of these drivers can explain the limited birch sapling growth in our study, where the limited resources gained by individuals are preferentially allocated to the birch tissues (stem width and/or roots) to cope in an environment still considered stressful.

Overall, we observed tundra community shifts over time where the *β‐*diversity changes were consistent over all three metrics and were dominated by a response within the Control plots between years. However, in contrast to what we observed in α‐diversity, the tundra communities are not getting more similar to the forest in *β‐*diversity, we rather show that the Control plots shift in an opposing direction and are getting more similar to the Betula plots compared with that in 2006. Vegetation transitions where vegetation has shifted toward a graminoid‐dominated tundra have been reported to be driven by herbivory (Wal, 2006; Zimov et al., 1995). However, the long‐term impact of warming climate may override the herbivore effect through an increase in shrub abundance shifting the vegetation state (Vowles et al., 2017). Climate change has been predicted to cause idiosyncratic responses and therefore generates entirely new communities, rather than a movement of preexisting ecosystems (Molau, 1997; Wang et al., 2021). Shifts to novel communities after drastic climatic change are not uncommon throughout the history of the Arctic flora, as exemplified by the flora shifting from a dry steppe, forb‐dominated system before, to a shrub‐ and graminoid‐dominated system after the last glacial maximum (Willerslev et al., 2014). Thus, our results, though restricted to one valley, are broadly in line with the projected climate‐driven altitudinal vegetation changes in the forest–tundra ecotone. However, the changes are small and, despite some indicative results, together with a 10‐year time frame could be too short to result in any major vegetation transitions. Furthermore, the small number of birches available for sampling may not have had the precision required to observe small shifts in diversity if they did occur.

## 5 CONCLUSION

The expansion of birch forest is known to facilitate vegetation change through buffering stress on neighboring plants (Eränen & Kozlov, 2008; Mikola et al., 2018). The birch saplings found above the treeline at Latnjajaure, however, are growth‐limited and do not affect the surrounding plant diversity. The lack of growth in spite of regional climate warming is likely explained by herbivory, nitrogen limitation, or a reduction in snow cover, though their importance needs to be experimentally verified. Independent of the presence of birches, the surrounding tundra community structure is shifting. Its direction is unclear, though it does not show any indication of shifting toward the birch forest communities. Thus, more evidence from both observations (Hofgaard et al., 2009; Kullman, 2010; Løkken et al., 2020; Rees et al., 2020), experimental (Løkken et al., 2019; Speed et al., 2011) and modeling (Gustafson et al., 2021), suggests that future treeline advancements are more restricted than commonly expected, implying that the vegetation change may be slower than hitherto assumed.

## AUTHOR CONTRIBUTIONS

**Ruud Scharn:** Conceptualization (equal); data curation (equal); formal analysis (lead); investigation (supporting); methodology (lead); visualization (lead); writing – original draft (equal); writing – review and editing (lead). **Isabel S. Negri:** Conceptualization (equal); data curation (equal); investigation (equal); methodology (equal); writing – original draft (equal); writing – review and editing (supporting). **Maja K. Sundqvist:** Conceptualization (equal); data curation (equal); writing – review and editing (supporting). **Jørn O. Løkken:** Investigation (supporting); writing – review and editing (supporting). **Christine D. Bacon:** Investigation (supporting); writing – review and editing (supporting). **Alexandre Antonelli:** Investigation (supporting); writing – review and editing (supporting). **Annika Hofgaard:** Conceptualization (equal); funding acquisition (supporting); resources (supporting); supervision (supporting); writing – review and editing (supporting). **R. Henrik Nilsson:** Investigation (supporting); writing – review and editing (supporting). **Robert G. Björk:** Conceptualization (equal); investigation (supporting); project administration (lead); resources (lead); supervision (lead); writing – original draft (equal); writing – review and editing (equal).

## CONFLICT OF INTEREST

The authors state that they have no conflicting interests.

## Data Availability

Vascular plant composition data, R data cleaning scripts, and the JAGS code for the statistical models can be found in the Swedish National Data Service catalog (https://doi.org/10.5878/p826‐y513).
